# Improving the clinical accuracy and flexibility of the Alkaptonuria severity score index

**DOI:** 10.1002/jmd2.12290

**Published:** 2022-05-10

**Authors:** Harriet E. O. Cant, Iro Chatzidaki, Birgitta Olsson, Mattias Rudebeck, Jean‐Baptiste Arnoux, Richard Imrich, Lucy A. Eddowes, Lakshminarayan R. Ranganath

**Affiliations:** ^1^ Costello Medical London UK; ^2^ Garriguella AB Ekerö Sweden; ^3^ OnPoint Science AB Stockholm Sweden; ^4^ Hôpital Necker‐Enfants Malades Paris France; ^5^ Institute of Clinical and Translational Research Biomedical Research Centre, Slovak Academy of Sciences Bratislava Slovakia; ^6^ National Institute of Rheumatic Diseases Piešťany Slovakia; ^7^ Department of Clinical Biochemistry and Metabolic Medicine Liverpool University Hospitals NHS Foundation Trust Liverpool UK; ^8^ Institute of Ageing and Chronic Disease University of Liverpool Liverpool UK

**Keywords:** Alkaptonuria, composite measure, disease progression, nitisinone, resource‐limited

## Abstract

Alkaptonuria (AKU) is a rare genetic disorder where oxidised homogentisic acid accumulates in connective tissues, leading to multisystem disease. The clinical evaluation Alkaptonuria Severity Score Index (cAKUSSI) is a composite score that assesses the extent of AKU disease. However, some components assess similar disease features, are difficult to measure reliably or cannot be measured in resource‐limited environments. cAKUSSI data from the 4‐year SONIA 2 randomised controlled trial, which investigated nitisinone treatment in adults with AKU, were analysed (*N* = 125). Potentially biased or low‐information cAKUSSI measurements were identified using clinical and statistical input to create a revised AKUSSI for use in AKU research (cAKUSSI 2.0). Additionally, resource‐intensive measurements were removed to explore a flexible AKUSSI (flex‐AKUSSI) for use in low‐resource environments. Revised scores were compared to cAKUSSI in terms of correlation and how they capture disease progression and treatment response. Eight measurements were removed from the cAKUSSI to create the cAKUSSI 2.0, which performed comparably to the cAKUSSI in measuring disease extent, progression and treatment response. When removing resource‐intensive measurements except for osteoarticular disease, the flex‐AKUSSI was highly correlated with the cAKUSSI, indicating that they quantified disease extent similarly. However, when osteoarticular disease (measured using scans) was removed, the corresponding flex‐AKUSSI underestimated disease progression and overestimated treatment response compared to the cAKUSSI. Clinicians may use the cAKUSSI 2.0 to reduce time, effort and patient burden. Clinicians in resource‐limited environments may find value in computing a flex‐AKUSSI score, offering potential for future global registries to expand knowledge about AKU.

**Synopsis**

Measurements may be removed from the clinical evaluation Alkaptonuria Severity Score Index to reduce resource use and patient burden and to increase flexibility, while still obtaining clinically relevant measures of alkaptonuria disease extent, progression and treatment response.

## 1 INTRODUCTION

Alkaptonuria (AKU; OMIM 203500) is a rare, inherited metabolic disorder that prevents patients from fully breaking down homogentisic acid (HGA), a metabolite of tyrosine.^1^, ^2^ Oxidised HGA accumulates in various connective tissues (ochronosis), leading to a range of manifestations that differ between affected individuals.^3^, ^4^, ^5^ The extent of AKU disease is often assessed using a composite measure called the Alkaptonuria Severity Score Index (AKUSSI), thus allowing multisystem disease progression and treatment response to be quantified.^6^, ^7^ The clinical evaluation AKUSSI (cAKUSSI) has been used in recent research and consists of many components, including spine and joint manifestations along with other non‐rheumatological features of AKU (Table S1).^7^, ^8^, ^9^ However, some measurements for components of the cAKUSSI assess similar features of AKU or may be difficult to measure reliably, potentially introducing noise to the cAKUSSI. Further, some measurements cannot be performed in resource‐limited environments, where equipment or specialist clinical expertise may not be available or where waiting lists are long. Such a limitation of the cAKUSSI is important to address, as AKU is more prevalent in consanguineous communities, which often have underfunded health systems.^8^

SONIA 2 was a 4‐year, randomised controlled trial that investigated the effects of once‐daily nitisinone for the treatment of AKU in adults;^6^ nitisinone inhibits the homogentisate 1,2‐dioxygenase enzyme (enzyme commission number 1.13.11.5) responsible for producing HGA.^10^ In this study, 138 patients were randomly assigned (1:1) to receive either oral nitisinone 10 mg daily or no treatment. Results from the SONIA 2 trial, as well as the UK's National Alkaptonuria Centre (NAC), have demonstrated that once‐daily nitisinone is well tolerated and effective in reducing urinary excretion of HGA and slowing disease progression.^6^, ^11^

With nitisinone now approved as a treatment for AKU,^12^ improving the cAKUSSI as a flexible and reliable tool to measure disease progression and treatment response globally is important for monitoring the real‐world efficacy of nitisinone as well as any future treatments. A more flexible AKUSSI may be of particular relevance to registries and large cohort studies where the availability of equipment and clinical expertise is likely to vary across participating sites. Therefore, we used the larger and more geographically dispersed sample from SONIA 2 to explore and revise the cAKUSSI for use in two different settings, building on a previous exploration of the AKUSSI that used a smaller dataset from the NAC.^9^ Firstly, we considered research environments, where the revised score should best reflect disease progression and treatment response (‘cAKUSSI 2.0’), and secondly resource‐limited environments, where limited equipment, expertise and capacity restricts the number of measurable components, and where a flexible score could prove beneficial (‘flexible AKUSSI’ [flex‐AKUSSI]).

## 2 METHODS

### 2.1 Study data

Patients from the SONIA 2 trial who gave data‐sharing consent were included in our analyses. Patients had a confirmed diagnosis of AKU with clinical disease manifestation of any kind and increased HGA, and were at least 25 years of age; further details on SONIA 2 have been previously published.^6^ cAKUSSI data were collected at baseline and annually thereafter. Missing data were generally imputed using last observation carried forward (LOCF), similar to the approach used for the SONIA 2 trial primary analyses.^6^, ^8^ In cases where LOCF was inappropriate, alternative imputation approaches were used (Supplementary Methods).

### 2.2 Statistical analysis

Measurements of the cAKUSSI were removed to create revised scores (the cAKUSSI 2.0 and the flex‐AKUSSI). As resource availability likely varies by provider, flex‐AKUSSI refers to a variety of scores defined by the lack of a particular resource. The revised scores were compared to the cAKUSSI to assess whether similar information is captured regarding disease extent and progression, and treatment response. A description of these revised AKUSSI scores, as well as AKUSSI scores used in previous research, can be found in Table S2.

### 2.3 cAKUSSI 2.0

In order to create the cAKUSSI 2.0, cAKUSSI measurements were identified for removal using a combination of methods: (1) Authors' expert opinion was used to assess which components may be unreliable or inherently biased when measuring the extent of AKU disease; the authors included principal investigators and a senior clinical pharmacologist for SONIA 2. Authors' expert opinion was also used to validate measurements suggested for removal by the statistical methods described below. (2) Principal component analysis (PCA) was used to analyse the contribution of each measurement to the variation in cAKUSSI score data, and therefore its ability to distinguish between patients of different disease severities, as per Langford et al.^9^ PCA was performed separately at each timepoint and results were compared to determine consistency over time. Relative weights from the first four principal components were used to identify which cAKUSSI components contributed most to the variation in the data. cAKUSSI measurements that consistently contributed a small amount of variation to the overall cAKUSSI were considered low‐information with respect to disease progression and therefore removed. To investigate the consistency of results between treatment groups, secondary analyses stratifying patients by treatment were performed. (3) Longitudinal trends were assessed for each component using alluvial plots, to identify measurements with little variation over time or with a small overall contribution to the cAKUSSI.

### 2.4 Flex‐AKUSSI

The flex‐AKUSSI was formed by removing resource‐intensive measurements (measurements that are not assessed via patient or clinician questionnaire) in turn from the cAKUSSI, to reflect that clinicians may need to remove different measurements based on the availability of resource‐intensive instruments. While resource‐intensive components of the cAKUSSI (Table 1) would ideally be measured, in practice their availability may vary by healthcare provider. As such, the flex‐AKUSSI enables the user to include any combination of the resource‐intensive components of the cAKUSSI available to them.

**TABLE 1:** Resource‐intensive AKUSSI components and corresponding resources required

Resource‐intensive components	Resource required
Hip osteopenia	DEXA scan
Aortic sclerosis/stenosis	Transthoracic echocardiography
Prostate stones and renal stones^a^	Ultrasound
Hearing loss	Air conduction audiometric test
Osteoarticular disease (joint and spine)	PET‐CT or Tc99m MDP scan
Kyphosis and scoliosis	X‐ray, Cobb angles measured by a clinical expert
Eye and ear pigmentation	Medical photography, to be interpreted by a clinical expert
Eardrum pigmentation	Otoscope

a Measured using ultrasound and self‐report.

Abbreviations: DEXA, dual energy x‐ray absorptiometry; PET‐CT, positron emission tomography–computed tomography; Tc99m MDP, technetium‐99 m methyl diphosphonate.

The flex‐AKUSSI was compared to the cAKUSSI. Firstly, for each piece of equipment used for cAKUSSI measurements, a flex‐AKUSSI was created where the corresponding resource‐intensive measurement was removed from the cAKUSSI. Next, all resource‐intensive measurements were removed together, with only those that are questionnaire‐based retained, to create a questionnaire flex‐AKUSSI.

As eye and ear cartilage pigmentation can often be seen by the naked eye, and in order to reflect realistic clinical practice in the absence of medical photography, eye and ear pigmentation scores were replaced with a clinician questionnaire proxy score instead of being removed completely (Table S4). In SONIA 2, pigmentation was measured using medical photography and was graded as either none, slight or marked for each eye. Eye pigmentation was also scored separately in two locations for each eye (temporal and nasal). In our proxy score, pigmentation in each eye and ear was only graded as ‘none’ or ‘present’, and eye pigmentation was not scored by location as this may be difficult to do in practice (i.e. a score of ‘present’ was given for the whole eye if nasal *or* temporal pigmentation was present). If present, values corresponding to slight pigmentation were given (8 per eye, 2 per ear), as this was the most common value observed in SONIA 2, thereby halving the maximal pigmentation score (40 with medical photography vs. 20 using the proxy). Eardrum pigmentation was not included in the proxy score because the location makes assessment difficult without specialist expertise and equipment.

### 2.5 Comparing revised scores with the cAKUSSI

Change from baseline AKUSSI score versus time, stratified by treatment, was plotted for both the cAKUSSI and the revised AKUSSI versions (cAKUSSI 2.0 and flex‐AKUSSI). The revised scores were adjusted to be on approximately the same scale as the cAKUSSI, allowing direct comparison of the values (Supplementary Methods). Scores were adjusted based on the maximum score in the cAKUSSI and revised scores, which were calculated using observed maximums for components with no upper limit (e.g. there is no limit on how many fractures a patient may experience). This visual comparison was further aided by computation of Spearman's correlations between the cAKUSSI and revised AKUSSI scores for each timepoint, to assess correlation.

## 3 RESULTS

### 3.1 Study subjects

Of the 138 patients randomised in SONIA 2, 125 patients gave data‐sharing consent and were included in this analysis. Of these, 61 were in the nitisinone arm (10 mg daily) and 64 in the control arm (no treatment). cAKUSSI data were available at 12 (*n* = 121), 24 (*n* = 119), 36 (*n* = 119) and 48 (*n* = 108) months post‐baseline. Similar to the overall SONIA 2 study population, the proportion of females in our study was 36%, the mean age was 48 years and the mean baseline cAKUSSI was 82 (Table 2).^6^

**TABLE 2:** Demographic data and baseline characteristics of the N = 125 patients included in the analyses

Characteristic	Control (*n* = 64)	Nitisinone (*n* = 61)	All patients (*N* = 125)
Age, years	47.17 (9.96)	48.08 (11.03)	47.62 (10.46)
Sex			
Female	27 (42.19%)	18 (29.51%)	45 (36.00%)
Male	37 (57.81%)	43 (70.49%)	80 (64.00%)
Race			
White	62 (96.88%)	59 (96.72%)	121 (96.80%)
Asian	2 (3.12%)	1 (1.64%)	3 (2.40%)
Black	0 (0%)	1 (1.64%)	1 (0.80%)
Study centre			
United Kingdom	19 (29.69%)	18 (29.51%)	37 (29.60%)
Slovakia	29 (45.31%)	27 (44.26%)	56 (44.80%)
France	16 (25.00%)	16 (26.23%)	32 (25.60%)
cAKUSSI score			
cAKUSSI, total	78.61 (34.11)	85.57 (34.54)	82.01 (34.36)
cAKUSSI, minimum, maximum	12, 163	14, 152	12, 163
Clinical^1^	40.91 (21.50)	46.75 (22.35)	43.76 (22.03)
Spine rheumatology^a^	18.05 (9.61)	19.39 (10.28)	18.70 (9.92)
Non‐spine (joint) rheumatology^a^	19.66 (8.87)	19.43 (10.33)	19.54 (9.57)
cAKUSSI score, individual items			
*Non‐rheumatological features*			
Eye pigmentation	13.69 (9.34)	16.72 (8.90)	15.17 (9.22)
Ear pigmentation	3.88 (2.85)	4.23 (2.95)	4.05 (2.89)
Eardrum pigmentation	8.06 (5.47)	8.66 (5.08)	8.35 (5.27)
Prostate stones	1.31 (2.26)	1.77 (2.58)	1.54 (2.42)
Renal stones	1.62 (3.40)	3.08 (5.68)	2.34 (4.69)
Hip osteopenia	2.12 (2.04)	2.23 (2.22)	2.18 (2.13)
Fractures	2.62 (7.81)	2.36 (5.53)	2.50 (6.77)
Ruptures	3.75 (6.68)	3.28 (7.92)	3.52 (7.28)
Aortic stenosis/sclerosis	1.50 (2.96)	2.13 (3.46)	1.81 (3.22)
Hearing loss	2.34 (2.09)	2.30 (2.30)	2.32 (2.18)
*Non‐spine rheumatology*			
Joint pain	4.62 (3.22)	4.77 (3.00)	4.70 (3.11)
Joint osteoarticular disease	13.47 (6.34)	12.13 (6.59)	12.82 (6.47)
Arthroscopies	0.44 (1.04)	0.62 (1.49)	0.53 (1.27)
Joint replacements	1.12 (3.22)	1.90 (4.04)	1.50 (3.65)
Spine rheumatology			
Spine pain	4.75 (2.38)	4.59 (2.67)	4.67 (2.51)
Spine osteoarticular disease	11.81 (8.62)	13.25 (8.90)	12.51 (8.75)
Scoliosis	0.97 (1.23)	0.92 (1.19)	0.94 (1.21)
Kyphosis	0.52 (1.26)	0.64 (1.35)	0.58 (1.30)

a Included features are detailed in the Table S1.

*Note*: Continuous data are summarised using mean (standard deviation [SD]); categorical data are summarised using *n* (%).

Abbreviation: cAKUSSI, clinical evaluation Alkaptonuria Severity Score Index.

### 3.2 cAKUSSI 2.0

#### 3.2.1 Defining the cAKUSSI 2.0

Expert clinical opinion suggested removing the following measurements from the cAKUSSI, thus creating the cAKUSSI 2.0: (1) Ultrasound‐detected renal stones: Renal stones can move depending on a patient's posture, meaning ultrasound scans often either miss or double‐count stones. (2) Ultrasound‐detected and self‐reported prostate stones: The number of prostate stones identified through ultrasound is often unreliable due to double‐counting the stones. Additionally, this component can only be included in the scores of male patients, making summary assessments of mixed sex groups difficult. (3) Eardrum pigmentation: Assessing eardrum pigmentation is subjective and is often challenging due to the presence of ear wax. It also requires specialist equipment and expertise such as medical photography. Eardrum pigmentation was also expected to add little information in addition to the eye and ear pigmentation scores. (4) Hearing loss: Hearing loss is a common condition with ageing, making its contribution to assessing the extent of disease limited, especially longitudinally. Further, some SONIA 2 participants may have had inaccurate measurements due to the presence of ear wax. (5) Arthroscopies: The authors' clinical experience suggested that arthroscopies may be performed infrequently, and indications for arthroscopies may differ between rheumatology departments, making comparability of results more difficult across patients or departments. In addition, joint pain, which is often an indication for arthroscopy, is already measured in the cAKUSSI.

PCA results showed that the following components consistently contribute a small amount of variation to cAKUSSI data (Figure S1), suggesting they are low‐information components: prostate stones (ultrasound and self‐reported), renal stones (ultrasound and self‐reported), fractures, kyphosis and scoliosis. Although the PCA suggested removing fractures, this was considered inadvisable due to the large observed relative contribution of fractures to the cAKUSSI for some patients (at the end of follow‐up, SONIA 2 participants scored up to 48 in the cAKUSSI due to fractures). Additionally, removing the self‐reported renal stones measurement would have resulted in a complete omission of renal stones from the cAKUSSI 2.0; this was considered inadvisable due to the importance of stone‐related renal failure for AKU patients, which can lead to death.^8^, ^13^, ^14^ All other measurements were shown to contribute variation to the AKUSSI, suggesting they should be retained; the spine osteoarticular disease component was particularly informative (Figure S1).

Finally, the analysis of longitudinal trends showed that prostate stones (ultrasound and self‐reported), renal stones (ultrasound) and arthroscopies demonstrated little change over time and may therefore provide little information on disease progression (Figure S2). Joint pain and hearing loss components changed the most over time; however, the results for hearing loss may be biased by the unreliability of these measurements (Figure S2).

The final cAKUSSI 2.0 was thus created through removing the following measurements from the cAKUSSI: prostate stones (ultrasound and self‐reported), renal stones (ultrasound), arthroscopies, eardrum pigmentation, kyphosis, scoliosis and hearing loss. A table of components included in the cAKUSSI 2.0, and the corresponding maximum scores observed in SONIA 2, can be found in Table S3.

#### 3.2.2 Comparison to the cAKUSSI

**FIGURE 1:**
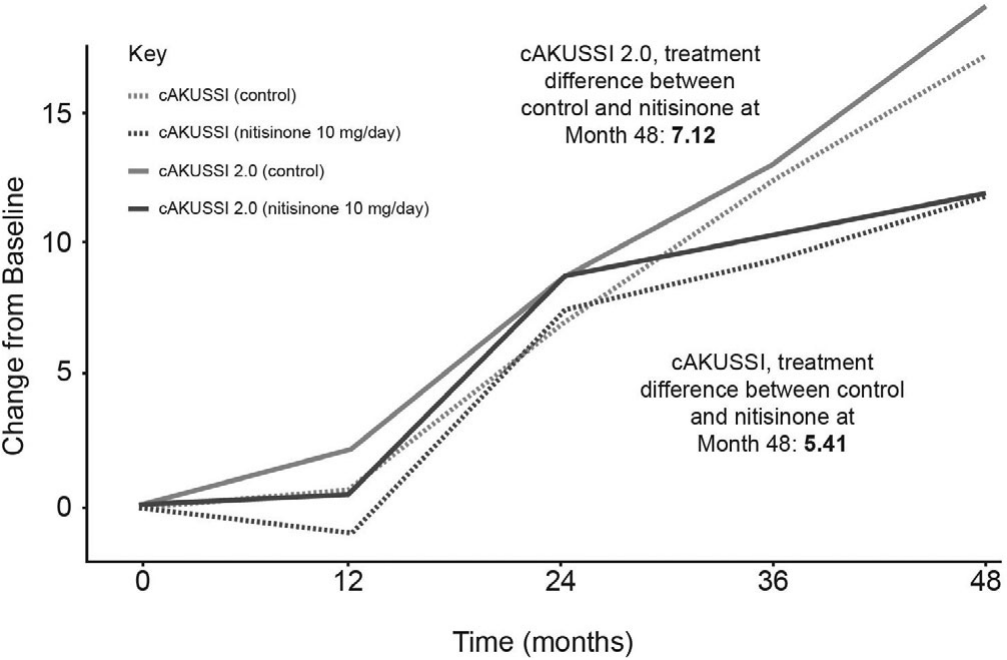
Change from baseline of the cAKUSSI 2.0 and cAKUSSI over time, nitisinone versus control. The cAKUSSI 2.0 was adjusted to be on the same scale as the cAKUSSI

The proposed cAKUSSI 2.0 was compared to the cAKUSSI. The cAKUSSI and cAKUSSI 2.0 were highly correlated at all timepoints (Spearman's correlations >0.97). Similar trends over time were seen in the cAKUSSI 2.0 and the cAKUSSI, implying that both captured disease progression similarly (Figure 1).

### 3.3 Flexible AKUSSI

#### 3.3.1 Removal of measurements by equipment

**FIGURE 2:**
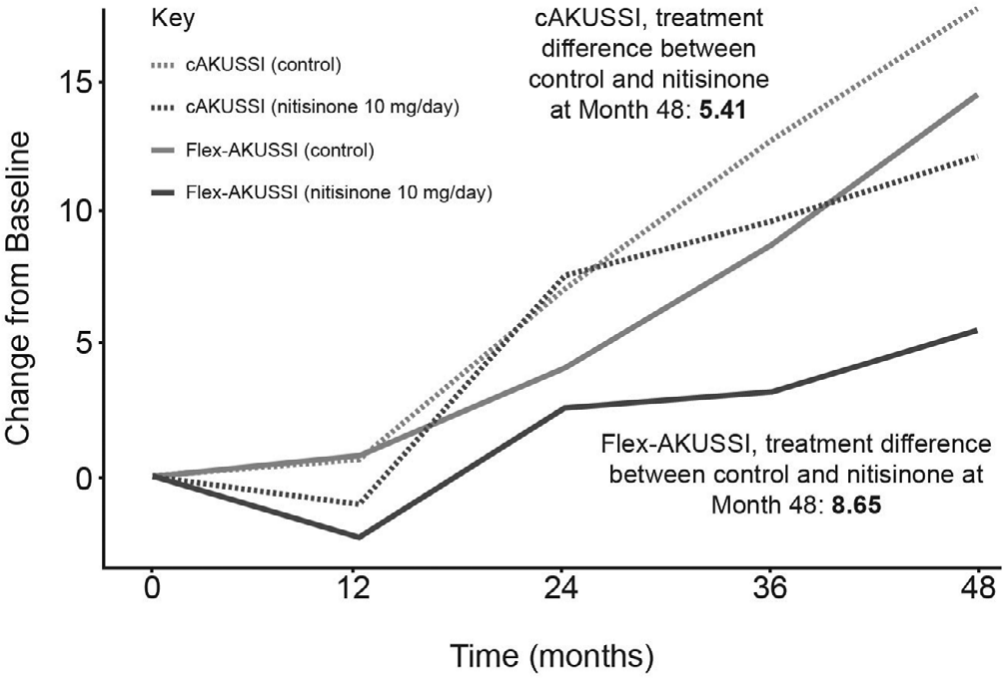
Change from baseline for the flex‐AKUSSI removing PET‐CT/Tc99m MDP scans and cAKUSSI over time, nitisinone versus control. The flex‐AKUSSI was adjusted to be on the same scale as the cAKUSSI

A flex‐AKUSSI was created for each piece of equipment used to calculate the cAKUSSI in which the corresponding resource‐intensive measurement was removed from the cAKUSSI score (Table 1). For all equipment except PET‐CT/Tc99m MDP scans, which measure joint and spine osteoarticular disease, the revised flex‐AKUSSI scores captured similar trends over time to the cAKUSSI in terms of disease progression and treatment response (Spearman's correlations >0.98 for each timepoint; Figure S5). When removing PET‐CT/Tc99m MDP scans, the flex‐AKUSSI showed a smaller percentage change over time, particularly for the nitisinone group, suggesting that it is underestimating disease progression compared with the cAKUSSI, and a larger divergence between the nitisinone and control arm, suggesting that it is overestimating treatment response (Figure 2) compared with the cAKUSSI. This result is in line with the observation that osteoarticular disease had a relatively large contribution to the overall cAKUSSI score (Table S3) and was shown to progress during SONIA 2 follow‐up (Table S6). Despite this, the flex‐AKUSSI lacking PET‐CT/Tc99m MDP scans was highly correlated with the cAKUSSI (Spearman's correlations >0.94 for each timepoint). Therefore, although the flex‐AKUSSI lacking PET‐CT/Tc99m MDP scans may measure disease progression and treatment response less accurately than the cAKUSSI, the flex‐AKUSSI captures similar trends to the cAKUSSI and may still serve as a useful tool for measuring extent of disease in settings where PET‐CT/Tc99m MDP scans are not available.

#### 3.3.2 Removal of all resource‐intensive measurements

**FIGURE 3:**
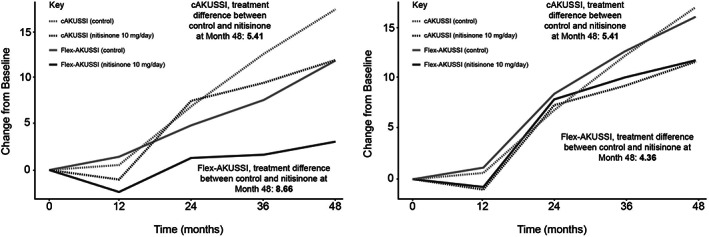
Change from baseline for the flex‐AKUSSI and cAKUSSI over time, nitisinone versus control. (A) Questionnaire flex‐AKUSSI with all resource‐intensive measurements removed; (B) Flex‐AKUSSI with all resource‐intensive measurements removed apart from PET‐CT/Tc99m MDP scan measurements. The flex‐AKUSSI was adjusted to be on the same scale as the cAKUSSI

A questionnaire flex‐AKUSSI score, in which all resource‐intensive measurements were removed (i.e. retaining questionnaire measurements only), was also analysed, to explore how the extent of disease may be captured in a setting with no specialist equipment available. A table of components included in the questionnaire flex‐AKUSSI, and the corresponding maximum scores observed in SONIA 2, can be found in Table S3. Due to the large relative weighting of the osteoarticular disease components, results were similar to those obtained when removing PET‐CT/Tc99m MDP scans only; the questionnaire flex‐AKUSSI and cAKUSSI were highly correlated at each timepoint (Spearman's correlations >0.8), implying that similar information is captured in both. However, the questionnaire flex‐AKUSSI appeared to underestimate disease progression and overestimate treatment response compared with the cAKUSSI (Figure 3). A second flex‐AKUSSI was therefore explored that removed all resource‐intensive measurements except for PET‐CT/Tc99m MDP scans. This adapted questionnaire flex‐AKUSSI performed well, capturing similar trends over time to the cAKUSSI in terms of disease progression and treatment response, and showing high correlation with the cAKUSSI at each timepoint (Spearman's correlations >0.9 for each timepoint; Figure 3).

## 4 DISCUSSION

Based on statistical and clinical input, the cAKUSSI 2.0 was formed by removing eight measurements (affecting 6/18 components) from the cAKUSSI (prostate stones [ultrasound and self‐reported], renal stones [ultrasound], arthroscopies, eardrum pigmentation, kyphosis, scoliosis and hearing loss; Table S1). The results showed that the cAKUSSI and the cAKUSSI 2.0 scores were highly correlated at all timepoints, indicating that both scores quantify the extent of disease similarly. In the analysis of change from baseline AKUSSI score, the cAKUSSI 2.0 captured comparable trends to the cAKUSSI for both nitisinone and control groups; this, along with the scores being highly correlated, suggests that researchers may reach similar conclusions regarding extent of disease, progression and treatment response using either metric. The similarity between the cAKUSSI and the cAKUSSI 2.0 was not unexpected due to the small number of measurements removed and the relatively small contribution of these to the cAKUSSI. Although the cAKUSSI 2.0 offered an improvement on the cAKUSSI by removing low‐information measurements, our results suggest that the cAKUSSI still captures the extent of AKU as a multisystem disease relatively effectively, as it was necessary to retain most components.

The reduced number of measurements used in the cAKUSSI 2.0 means that clinicians may measure extent of disease with fewer logistical considerations and reduced patient burden compared with the cAKUSSI. As such, the cAKUSSI 2.0 is proposed as the primary score to be used by clinicians. While measurements were removed from the cAKUSSI on the basis of unreliability or low information, clinicians may wish to continue taking these measurements in order to monitor AKU symptoms. For example, although prostate stones may not be suitable to include when researching disease extent, due to an inherent sex‐bias, they may still provide useful clinical insight for managing AKU for individual patients. Additionally, researchers in resource‐limited environments, where components particularly valuable for measuring disease progression may not be available, may still want to include self‐reported prostate stones and arthroscopies in calculating the AKUSSI.

Exploring the impact of fewer resources on measuring the extent of disease further, when resource‐intensive measurements were removed in turn from the cAKUSSI, we found that, with the exception of PET‐CT/Tc99m MDP scans (osteoarticular disease), the resulting flex‐AKUSSI scores (one flex‐AKUSSI per piece of equipment) performed well as a measure of disease extent. A flex‐AKUSSI that simultaneously removed all resource‐intensive measurements, except for PET‐CT/Tc99m MDP scans, was also explored (6/18 components removed; Table S1). This flex‐AKUSSI was highly correlated with the cAKUSSI at all timepoints, and an analysis of change from baseline AKUSSI score showed that both captured disease progression and treatment response similarly. Thus, with the exception of PET‐CT/Tc99m MDP scans, healthcare providers with limited resources may still effectively measure extent of AKU disease with minimal loss of information. However, resource‐intensive measurements should be captured where possible to provide the greatest accuracy.

In the absence of PET‐CT/Tc99m MDP scans however, while scores were still correlated with the cAKUSSI, the use of a flex‐AKUSSI may underestimate disease extent and progression when compared with the cAKUSSI, due to the importance of osteoarticular disease for patients with AKU (reflected by the number of cAKUSSI measurements assessing osteoarticular disease). Thus, to measure the overall extent of disease and disease progression, the inclusion of osteoarticular measurements is important. Several alternative less resource‐intensive measures could be considered in place of PET‐CT/Tc99m MDP scans; for example, the use of X‐ray imaging (more readily available than PET‐CT/Tc99m MDP scans), instruments that assess pain (typically self‐reported questionnaires) or instruments that measure the impact of disease on daily living (e.g. HAQ‐DI) could be used as proxies. While pain has been shown to correlate with PET‐CT‐assessed osteoarticular disease in a study of patients enrolled at the NAC,^15^ suggesting it could be a suitable proxy for PET‐CT/Tc99m MDP scans, pain and PET‐CT/Tc99m MDP scan scores were poorly correlated for SONIA 2 participants (data not shown). However, in the cAKUSSI, pain is assessed as a binary variable (pain/no pain), and a more granular assessment of pain may have yielded different results. Further research is needed to explore suitable alternatives to PET‐CT/Tc99m MDP scans for healthcare providers that are not able to perform these scans. Further research could also explore suitable less resource‐intensive alternatives for other measurements of the cAKUSSI, such as phone apps to measure hearing loss or using a stethoscope instead of an echocardiogram to assess the presence of aortic stenosis.

Our work suggests that the absence of equipment used for the cAKUSSI should not hinder at least a partial assessment of AKU disease status. This may be valuable for future research where data from multiple centres with varying equipment could be combined, for example in an upcoming global registry of AKU where resources may be particularly limited for those in low‐ and middle‐income countries. In addition to between‐centre variation in AKU assessment and monitoring, there could also be variation within centres or for the same patient over time (e.g. if a centre changes clinical practice or a patient relocates). Ideally, researchers should use the same metric to compare patients for greatest accuracy. However, if improvements are necessary and clinically validated, replacing an existing metric with an updated version may be preferable to persisting with a previous version with limited accuracy. In addition, modifications may be made to adjust the revised AKUSSI scores (cAKUSSI 2.0 or flex‐AKUSSI) to be on the same scale as the cAKUSSI scores and our work demonstrates that these alternatives are well correlated, likely due to the underlying ochronosis driving many manifestations of AKU. Making such adjustments may allow the aggregation and comparison of data on AKU disease extent obtained using different combinations of equipment without the need to recalculate scores, enabling greater flexibility for data sets around the world. Our results support the cAKUSSI as an effective measure of the extent of AKU as a multisystem disease, as most components were necessary to retain.

Strengths of our work include using both clinical and statistical input to critically assess the utility of each cAKUSSI measurement, which overlapped well; out of the six measurements indicated for removal by clinical input, the statistical analysis supported the removal of five, highlighting that some of the limitations of these measures in clinical practice are reflected in the observed data. Further, our analyses were performed using a larger and more geographically dispersed sample than previous assessments of the cAKUSSI, and included both nitisinone‐treated and untreated patients, allowing assessment of how well alternative AKUSSI versions captured treatment response.^9^

A limitation of the analyses is the small sample size used compared with the number of cAKUSSI measurements, which may have limited the use of PCA to accurately determine low‐information scores. However, sample size limitations are common in rare disease research, where study population sizes are inherently small. Data from SONIA 2 were available for 4 years of follow‐up whereas AKU is a life‐long condition; as a result, conclusions on the cAKUSSI and modifications to it may have differed if the analyses had covered a longer follow‐up period that is more representative of the chronic nature of AKU and that captured potential long‐term treatment effects. Other limitations of our analyses include the absence of other AKU disease measures against which findings could be validated. Also, since disease extent is not directly observable, it is unclear how accurately the revised scores assessed true clinical status, a common challenge when measuring heterogeneous diseases using a composite score. However, the strong positive correlation of the revised scores with the cAKUSSI may reassure clinicians that the cAKUSSI 2.0 and the flex‐AKUSSI measure disease extent similarly to the cAKUSSI.

The cAKUSSI 2.0 and flex‐AKUSSI would ideally be used without the need for updates, to aid comparability of data over different studies and to avoid confusion among clinicians. However, future work to further optimise the AKUSSI tool could focus on ensuring that the weighting assigned to each component is fully reflective of its clinical relevance or capacity to assess disease progression or treatment response over time; this would likely impact the PCA results, altering the relative importance of each component in the overall cAKUSSI score, as well as the impact of removing measurements. This is particularly relevant for pigmentation components, which are weighted highly in observed cAKUSSI data (Table 2). As pigmentation manifestations were particularly affected by nitisinone treatment in SONIA 2 and other cohorts, any changes in weighting may alter how the cAKUSSI and its modifications reflect treatment response.^6^, ^16^ Therefore, researchers should consider the relative weighting of pigmentation components when using revised AKUSSI scores. Future research could also explore the use of age adjustment after analysing how the cAKUSSI performs with age, given that AKUSSI components such as hearing loss, spine features and hip or knee replacements correlate with age in the general population. Further, the addition of new components could be explored; measures such as ‘range of motion’ were identified as being clinically meaningful for measuring disease progression and treatment response in the SONIA 2 trial but are not currently included in the AKUSSI.^6^

## 5 CONCLUSIONS

The cAKUSSI 2.0, in which measurements known to be difficult to measure, low‐information or inherently biased were removed, performed comparably to the cAKUSSI in terms of disease progression and treatment response. Clinicians and researchers may wish to use this revised score to save time, effort and resources and reduce the burden to patients. Clinicians and researchers working in resource‐limited environments can be reassured that calculating a flex‐AKUSSI score without certain resource‐intensive measurements is still valuable for monitoring AKU disease progression, although assessing osteoarticular disease was important. These alternative AKUSSI measures offer potential for use in global registries to gain a good approximation of disease as researchers look to expand AKU knowledge in an era of approved treatments for AKU.

## CONFLICT OF INTEREST

Harriet E. O. Cant, Iro Chatzidaki and Lucy A. Eddowes are employees of Costello Medical. Birgitta Olsson and Mattias Rudebeck were shareholders of Sobi at the time of these analyses, and were employees of Sobi at the initiation of these analyses. In the last 5 years, Lakshminarayan R. Ranganath has received honoraria and consulting fees from Sobi. Jean Baptiste Arnoux and Richard Imrich declare that they have no conflict of interest.

## INFORMED CONSENT

All procedures followed in SONIA 2 were in accordance with the ethical standards of the responsible committee on human experimentation (institutional and national) and with the Helsinki Declaration of 1975, as revised in 2000 (5). Informed consent was obtained from all patients in SONIA 2, and only those patients who had given data‐sharing consent were included in this post hoc analysis. Independent ethics committees at each centre (Royal Liverpool University Hospital, Liverpool, UK; Hôpital Necker‐Enfants Malades, Paris, France; National Institute of Rheumatic Diseases, Piešťany, Slovakia) approved the SONIA 2 study.

## ANIMAL RIGHTS

Animal subjects were not used in this study.

## Supporting information


**Appendix S1** Supporting information

## Data Availability

Data will be made available on reasonable request.
