# Supplementary material for: Improving the clinical accuracy and flexibility of the Alkaptonuria severity score index
Source: JIMD Rep. 2022 May 10;63(4):361–70. doi: 10.1002/jmd2.12290 (PMC9259391; doi:10.1002/jmd2.12290)
Supplement: Supplementary file 1 — Appendix S1 Supporting information [file JMD2-63-361-s001.docx]

SUPPLEMENTARY MATERIAL – Improving the clinical accuracy and flexibility of the Alkaptonuria Severity Score Index

**Table of Contents**

[Supplementary Methods 2](#_Toc93568571)

[The AKUSSI 2](#_Toc93568572)

[Study data 5](#_Toc93568573)

[Statistical analysis 6](#_Toc93568574)

[Pigmentation proxy scores 6](#_Toc93568575)

[Adjustment factors and equations to adjust revised AKUSSI scores to be on the cAKUSSI scale 7](#_Toc93568576)

[Supplementary Results 8](#_Toc93568577)

[Missing data 8](#_Toc93568578)

[Principal component analysis 9](#_Toc93568579)

[Longitudinal trends 10](#_Toc93568580)

[Flex-AKUSSI 17](#_Toc93568581)

[Contribution of osteoarticular disease 18](#_Toc93568582)

# Supplementary Methods

## The AKUSSI

Supplementary Table 1: cAKUSSI score used in SONIA 2

| **Feature** | | **Score** | **Feature** | | | **Score** | **Method of Assessment** | **No. of components** |
| --- | --- | --- | --- | --- | --- | --- | --- | --- |
| **NON-RHEUMATOLOGIC FEATURES** | | | | | | | | |
| **Eye pigment** | | | | | | | Medical Photography | 1 |
| Right eye (nasal) | Slight  Marked | 4  8 | Left eye (nasal) | Slight  Marked | | 4  8 |  |  |
| Right eye (temporal) | Slight  Marked | 4  8 | Left eye (temporal) | Slight  Marked | | 4  8 |  |  |
| **Ear pigment** | | | | | | | Medical Photography | 1 |
| Right ear | Slight  Marked | 2  4 | Left ear | Slight  Marked | | 2  4 |  |  |
| **Stones** | | | | | | | Ultrasound and self-report | 2  (1 prostate, 1 renal) |
| Prostate stones | Per episode | 4 | Renal stones | Per episode | | 4 |  |  |
| **Musculoskeletal** | | | | | | | DEXA scan | 1 |
| Osteopenia hip | T-scores |  |  | | | |  |  |
|  | -1.0 to -1.7 | 2 |  |  |  |  |  |  |
|  | -1.8 to -2.4 | 4 |  |  |  |  |  |  |
|  | <-2.5 | 6 |  |  |  |  |  |  |
| Adult fracture | Per fracture | 8 | Rupture (muscle/  ligament/  tendon) | Per rupture | | 8 | Self-report | 2 |
| **Heart** | | | | | | | Transthoracic echocardio-graphy | 1 |
| Aortic sclerosis | | 4 | Aortic valve stenosis | Mild  Moderate  Severe | | 8  10  12 |  |  |
| **Hearing and eardrum** | | | | | | | Hearing loss: Audiometric test  Dark eardrum: Otoscope | 2 |
| Hearing loss | Maximum dB loss per ear |  | Dark eardrum | Per ear | | 6 |  |  |
|  | 21–35 (mild) | 1 |  |  |  |  |  |  |
|  | 36–60 (moderate) | 2 |  |  |  |  |  |  |
|  | >60 (severe) | 4 |  |  |  |  |  |  |
| **NON-SPINE RHEUMATOLOGY** | | | | | | | | |
| Clinical joint pain  (hips, knees, ankles, feet, shoulders, elbows, wrists & hands - right and left sides) | | | | | | 1 per joint (max 14) | Self-report | 1 |
| Non-spine osteoarticular disease  (hips, knees, ankles, feet, shoulders, elbows, wrists & hands - right and left sides) | | | | | | 2 per joint (max 28) | Tc99m MDP scan (or PET-CT) | 1 |
| Arthroscopies | | | | | | 2 each | Self-report | 1 |
| Joint replacements | | | | | | 4 each | Self-report | 1 |
| **SPINE RHEUMATOLOGY** | | | | | | | | |
| Clinical spinal pain  (cervical, thoracic, lumbar, sacroiliac) | | | | | | 2 per area (max 8) | Self-report | 1 |
| Osteoarticular disease of the spine  (pubic symphysis, ribs, sacroiliac, lumbar, thoracic, cervical) | | | | | | 2 per area (max 24) | PET-CT or Tc99m MDP scan | 1 |
| Kyphosis | Cobb angles  45⁠–60  >60 | 3  6 | Scoliosis | | Cobb angles  5–20  21–30  >30 | 2  4  6 | X-ray | 2 |

cAKUSSI: clinical evaluation Alkaptonuria Severity Score Index; DEXA: dual energy X-ray absorptiometry; PET-CT: positron emission tomography–computed tomography; Tc99m MDP: technetium-99m methyl diphosphonate.

Supplementary Table 2: AKUSSI versions in the literature

| **AKUSSI Version** | **Description** | **Use** |
| --- | --- | --- |
| Clinical evaluation AKUSSI | First version of the comprehensive measure of AKU disease extent | Introduced in Cox & Ranganath ^1^ |
| Questionnaire AKUSSI (qAKUSSI) | A version of the above using questionnaire components only |  |
| cAKUSSI | Slightly modified version of the above that better reflects longitudinal changes; used in SONIA 2 | Used in SONIA 2 ^2^ |
| Modified AKUSSI (mAKUSSI) | cAKUSSI (as used in SONIA 2) without pigmentation components |  |
| cAKUSSI 2.0 | Revised version of the  SONIA 2 cAKUSSI with low-information and unreliable measurements removed | Introduced and discussed in this paper |
| Flexible AKUSSI (flex-AKUSSI) | A series of simplified versions of the SONIA 2 cAKUSSI for environments without full resource (e.g. including questionnaire measurements only). The flexibility of this score refers to resource availability varying across different healthcare providers; clinicians may remove measurements according to their locally available resources.  The flex-AKUSSI scores in this paper were developed by removing resource-intensive measurements in turn from the cAKUSSI. |  |
| Questionnaire flexible AKUSSI (questionnaire flex-AKUSSI) | A simplified version of the SONIA 2 cAKUSSI developed in this paper that includes questionnaire measurements only, for use in environments without full resource. |  |

AKUSSI: Alkaptonuria Severity Score Index.

Supplementary Table 3: Summary of cAKUSSI components included in the cAKUSSI 2.0 and questionnaire flex-AKUSSI scores, and respective weights

| **cAKUSSI component** | **SONIA 2 maximum value** | **cAKUSSI 2.0** | **Questionnaire flex-AKUSSI^*^** |
| --- | --- | --- | --- |
| Eye pigmentation  (temporal and nasal) | 16/eye | Y | Y (proxy score; 8/eye)^†^ |
| Ear pigmentation | 4/ear | Y | Y (proxy score; 2/ear)^†^ |
| Prostate stones (ultrasound) | 8^§^ | N | N |
| Prostate stones (self-reported) | 20^§^ | N | Y |
| Renal stones (ultrasound) | 12^§^ | N | N |
| Renal stones (self-reported) | 32^§^ | Y | Y |
| Osteopenia hip | 6 | Y | N |
| Adult fracture | 48 | Y | Y |
| Ruptures  (tendon, ligament and muscle) | 56^§^ | Y | Y |
| Aortic valve sclerosis/stenosis | 12 | Y | N |
| Hearing impairment | 4/ear | N | N |
| Dark eardrum | 6/ear | N | N |
| Clinical joint pain  (hips, knees, ankles, feet, shoulders, elbows, wrists & hands) | 14 | Y | Y |
| Joint osteoarticular disease  (Hips, knees, ankles, feet, shoulders, elbows, wrists & hands) | 28 | Y | N |
| Arthroscopies | 6^§^ | N | Y |
| Joint replacements | 24^§^ | Y | Y |
| Clinical spinal pain  (cervical, thoracic, lumbar, sacroiliac) | 8 | Y | Y |
| Spine osteoarticular disease  (pubic symphysis, ribs, sacroiliac, lumbar, thoracic, cervical) | 24 | Y | N |
| Kyphosis | 6 | N | N |
| Scoliosis | 6 | N | N |
| Maximum potential score based on observations in our cohort | 370^§^ | 292 | 228 |

^*^Removing all resource-intensive measurements (retaining questionnaire measurements only). Measurements marked with a ‘N’ (excluded) are those considered to be resource-intensive. ^†^In the flex-AKUSSI, ear and eye pigmentation scores (excluding eardrum pigmentation) are replaced with a proxy score instead of being removed completely. In the proxy score, the maximal value is halved. ^§^Scores are assigned per event for fractures, ruptures, stones, arthroscopies and joint replacements. As there is no upper limit on the number of events, the maximal score was taken based on the observed data in SONIA 2 (using the observed maximum across all timepoints).

cAKUSSI: clinical evaluation Alkaptonuria Severity Score Index.

## Study data

A threshold of 20 Cobb angles was used to define moderate scoliosis in the cAKUSSI used in SONIA 2 (Supplementary Table 1).^2^ Based on authors’ expert opinion, a threshold of 10 was used in this analysis.

Missing data were imputed using last observation carried forward (LOCF) as the default method of imputation. To limit the extent of imputation in the analyses and the assumptions associated with it, values for missing visits were not imputed. In cases where missingness was considered informative, or LOCF could not be performed, alternative imputation approaches, verified by authors’ expert opinion, were considered:

- For patients with no hearing loss data due to deafness, a score associated with maximum hearing loss was used.
- For all patients with missing self-reported prostate (/renal) stones, ultrasound data were available and indicated 0 stones. Similarly, for all patients with missing ultrasound data for prostate (/renal) stones, self-reported data were available and indicated 0 stones. As there is no supposed clinical reason for missingness, and no reason why these examinations should not be performed, missing values were imputed as 0 based on clinical input.
- For patients with missing values due to joint replacements (e.g. hip osteopenia missing due to no native hips), a LOCF approach was used to reflect the severity of disease in the joint that may have led to a replacement.
- For patients with other missing data at baseline, where LOCF was not possible, the corresponding measurement was not included when calculating the AKUSSI score at that timepoint, as per the approach in SONIA 2.

## Statistical analysis

The cAKUSSI scores (rather than raw input data, e.g. Cobb angles) were used for the principal component analysis (PCA). Additionally, left and right constituents were summed prior to the analysis (e.g. left and right hearing loss was summed, and total hearing loss was used in all analyses), and muscle, ligament and tendon ruptures were combined in a single component.

All analyses were conducted using R statistical software (Version 4.1.0).^3^ Principal component analyses were conducted using *prcomp* from the *stats* package.^3^ Alluvial plots assessing longitudinal trends were created using the *ggalluvial and ggplot2* packages.^4, 5^

## Pigmentation proxy scores

Supplementary Table 4: Proxy scores used for eye and ear pigmentation in the absence of medical photography

|  | **Original Score** | | **Max/ Patient** | **Proxy Score** | | **Max/ Patient** |
| --- | --- | --- | --- | --- | --- | --- |
| **Eye**  Each eye is scored | Temporal pigmentation | Nasal pigmentation | 16/eye | Visible pigmentation anywhere in the eye | | 8/eye |
|  | None: 0 | None: 0 |  | Yes: 8 | No: 0 |  |
|  | Slight: 4 | Slight: 4 |  |  |  |  |
|  | Marked: 8 | Marked: 8 |  |  |  |  |
| **Ear**  Each ear is scored | Cartilage pigmentation via medical photography | | 4/ear | Visible cartilage pigmentation | | 2/ear |
|  | None: 0 | |  | Yes: 2 | No: 0 |  |
|  | Slight: 2 | |  |  |  |  |
|  | Marked: 4 | |  |  |  |  |

## Adjustment factors and equations to adjust revised AKUSSI scores to be on the cAKUSSI scale

To ensure that comparisons between the revised AKUSSI scores (cAKUSSI 2.0 and flex-AKUSSI) and the cAKUSSI were on a comparable scale, the revised AKUSSI scores were adjusted to be on the cAKUSSI scale. The following formula was used:

$$Rescaled revised AKUSSI=\frac{Revised AKUSSI}{Maximum revised AKUSSI}\times Maximum cAKUSSI$$

The maximum scores were calculated by summing the maximum point contribution per component (Supplementary Table 3).

# Supplementary Results

## Missing data

Supplementary Table 5: Number of patients with missing data at each timepoint for measurements with missingness present

| **Measurement** | **Baseline**  **(N=125)** | **Month 12**  **(N=121)** | **Month 24**  **(N=119)** | **Month 36**  **(N=119)** | **Month 48**  **(N=108)** |
| --- | --- | --- | --- | --- | --- |
| **Osteoarticular disease** | | | | | |
| Hip, right | 5 | 4 | 14 | 22 | 17 |
| Hip, left | 3 | 5 | 14 | 22 | 17 |
| Knee, right | 13 | 12 | 23 | 32 | 28 |
| Knee, left | 12 | 13 | 23 | 31 | 25 |
| Ankle, right | 2 | 2 | 9 | 14 | 8 |
| Ankle, left | 2 | 2 | 9 | 14 | 8 |
| Foot, right | 2 | 2 | 9 | 14 | 8 |
| Foot, left | 2 | 2 | 9 | 14 | 8 |
| Shoulder, right | 5 | 5 | 13 | 18 | 12 |
| Shoulder, left | 5 | 5 | 13 | 18 | 13 |
| Elbow, right | 2 | 2 | 9 | 14 | 8 |
| Elbow, left | 2 | 2 | 9 | 14 | 8 |
| Wrist and hand, right | 2 | 2 | 9 | 14 | 8 |
| Wrist and hand, left | 2 | 2 | 9 | 14 | 8 |
| Cervical spine | 2 | 2 | 9 | 14 | 8 |
| Lumbar spine | 2 | 2 | 9 | 14 | 8 |
| Thoracic spine | 2 | 2 | 9 | 14 | 8 |
| Sacroiliac | 2 | 2 | 9 | 14 | 8 |
| Ribs | 2 | 2 | 9 | 14 | 8 |
| Pubic symphysis | 2 | 2 | 9 | 14 | 8 |
| **Hearing loss** | | | | | |
| 0.5, 1, 2 and 4 kHz, right | 2 | 1 | 1 | 3 | 1 |
| 8 kHz, right | 4 | 1 | 3 | 3 | 3 |
| 0.5, 1, 2 and 4 kHz, left | 2 | 1 | 1 | 3 | 1 |
| 8 kHz, left | 4 | 2 | 3 | 3 | 1 |
| **Other** | | | | | |
| Eardrum pigmentation, right | 3 | 2 | 0 | 3 | 5 |
| Eardrum pigmentation, left | 3 | 2 | 0 | 3 | 5 |
| Prostate stones, self-report^*^ | 1 | 0 | 0 | 0 | 1 |
| Prostate stones, ultrasound^*^ | 0 | 4 | 2 | 3 | 1 |
| Renal stones, self-report | 1 | 0 | 0 | 0 | 0 |
| Hip osteopenia | 3 | 5 | 8 | 11 | 15 |
| Arthroscopies | 1 | 0 | 0 | 0 | 0 |
| Kyphosis | 0 | 0 | 1 | 0 | 0 |

^*^Prostate stones were not considered as missing for female patients. Measurements not listed in this table had no missing data.

## Principal component analysis

Supplementary Figure 1: Principal component analysis results heatmap


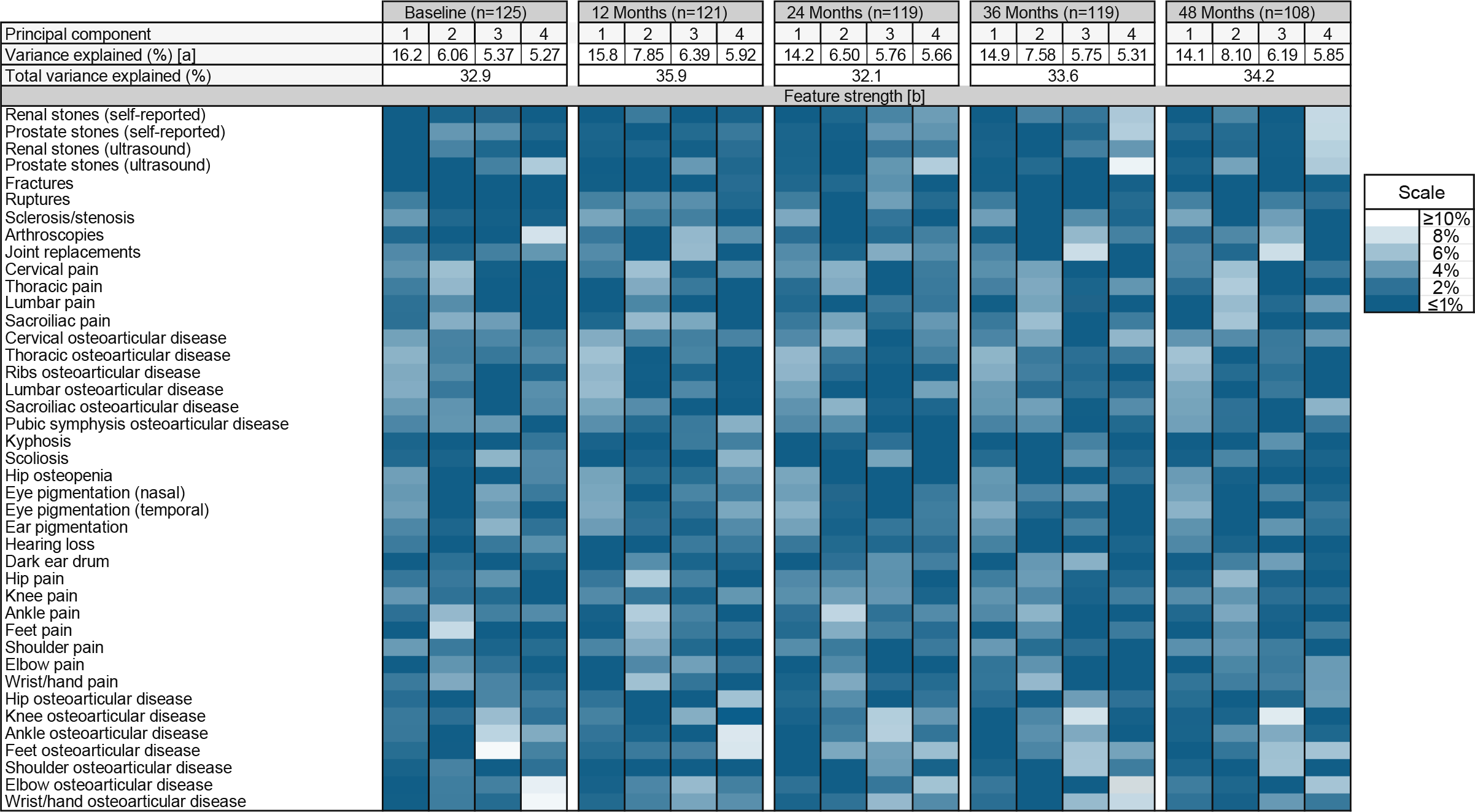


^a^Proportion of the variance of the data at that visit explained by the given principal component; only the first four principal components are shown; ^b^Proportion of the individual principal component’s variance explained by the given feature. Lighter rectangles denote a larger proportion of the variance; darker rectangles are features which do not contribute much to the variance.

## Longitudinal trends

The alluvial plots (Supplementary Figures 2–4) show changes in features of the cAKUSSI score for measurements over time. Each bar represents a different visit, and the thickness of the streams that connect each bar represent the number of patients whose scores increased, decreased or stayed the same between visits. The plots thus show which measurements make a small overall contribution to the AKUSSI, or have little variation over time.

Supplementary Figure 2: Alluvial plots showing changes in non-rheumatological features of the AKUSSI score for components over time, nitisinone vs control
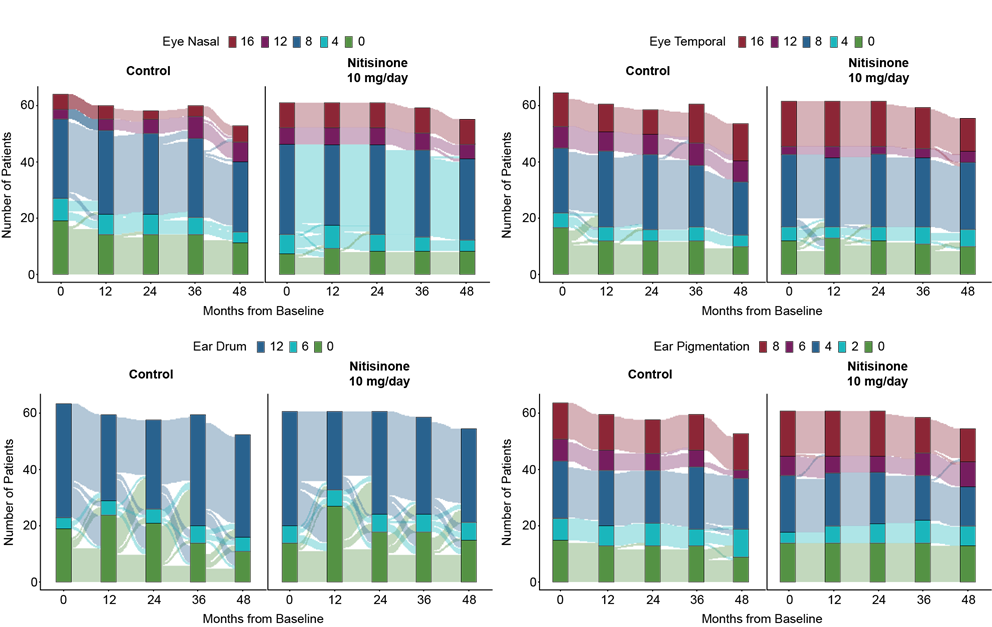


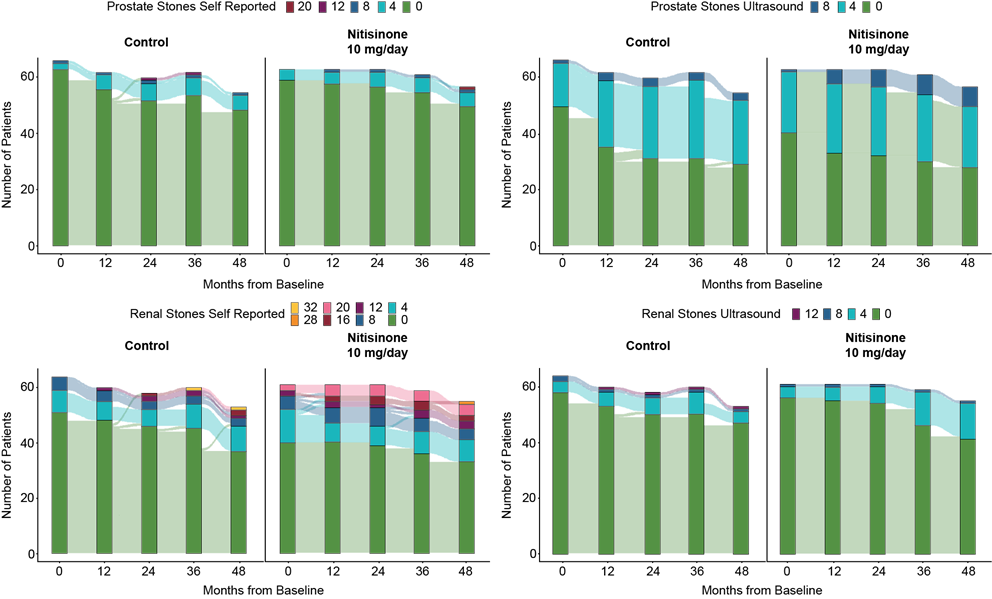


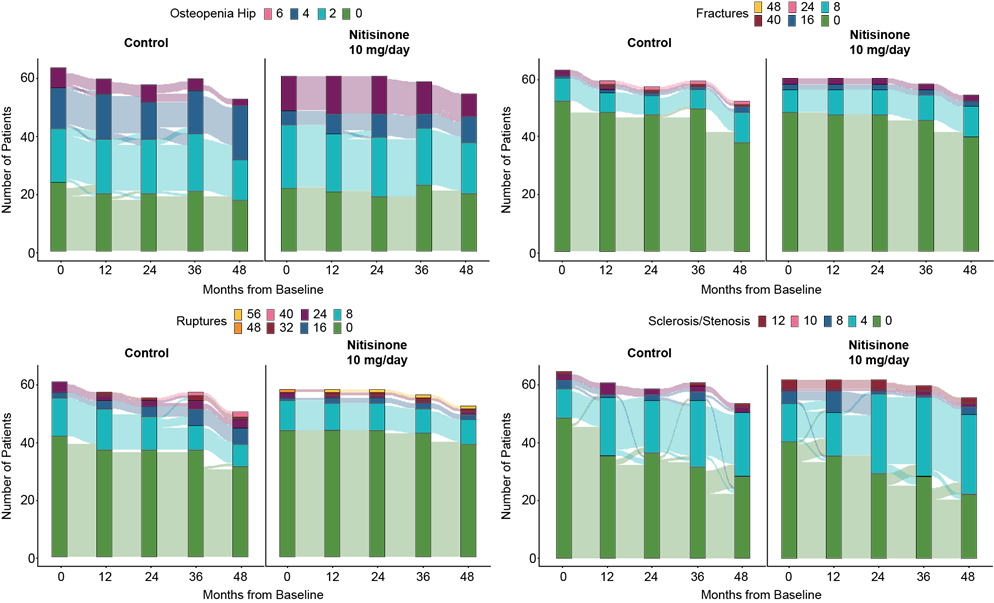

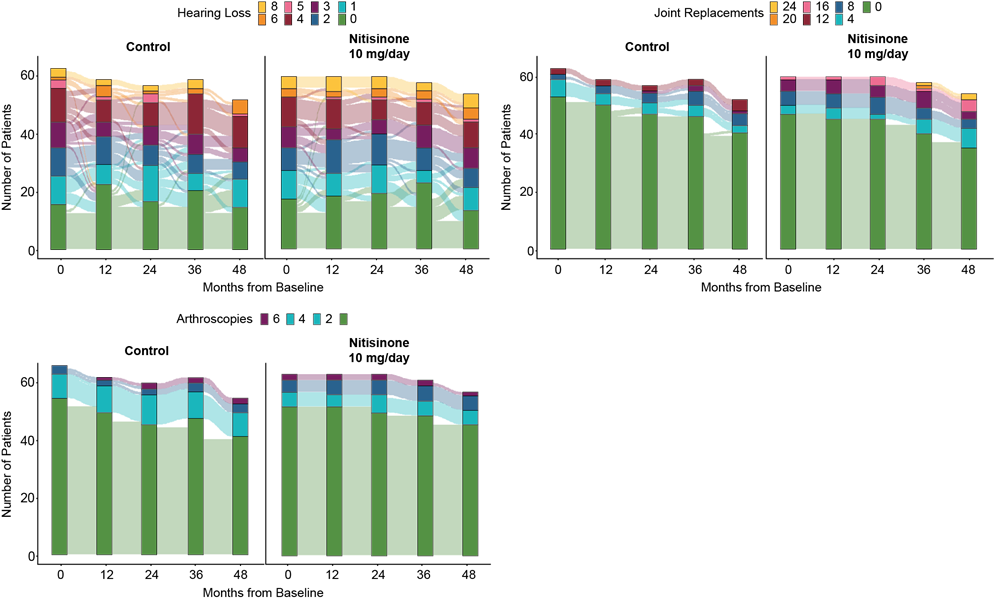


AKUSSI: Alkaptonuria Severity Score Index.

Supplementary Figure 3: Alluvial plots showing changes in joint rheumatology features of the AKUSSI score for components over time, nitisinone vs control


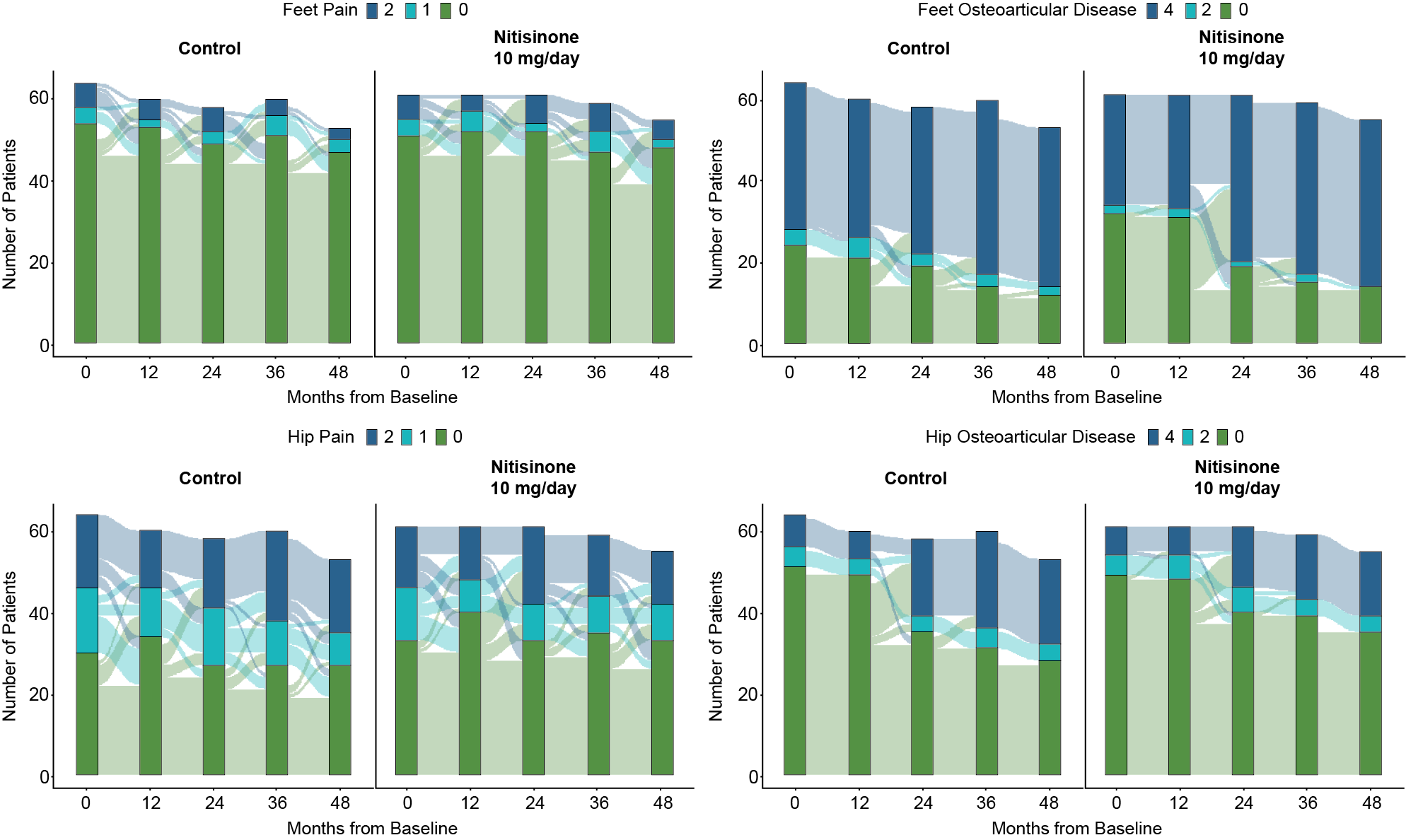

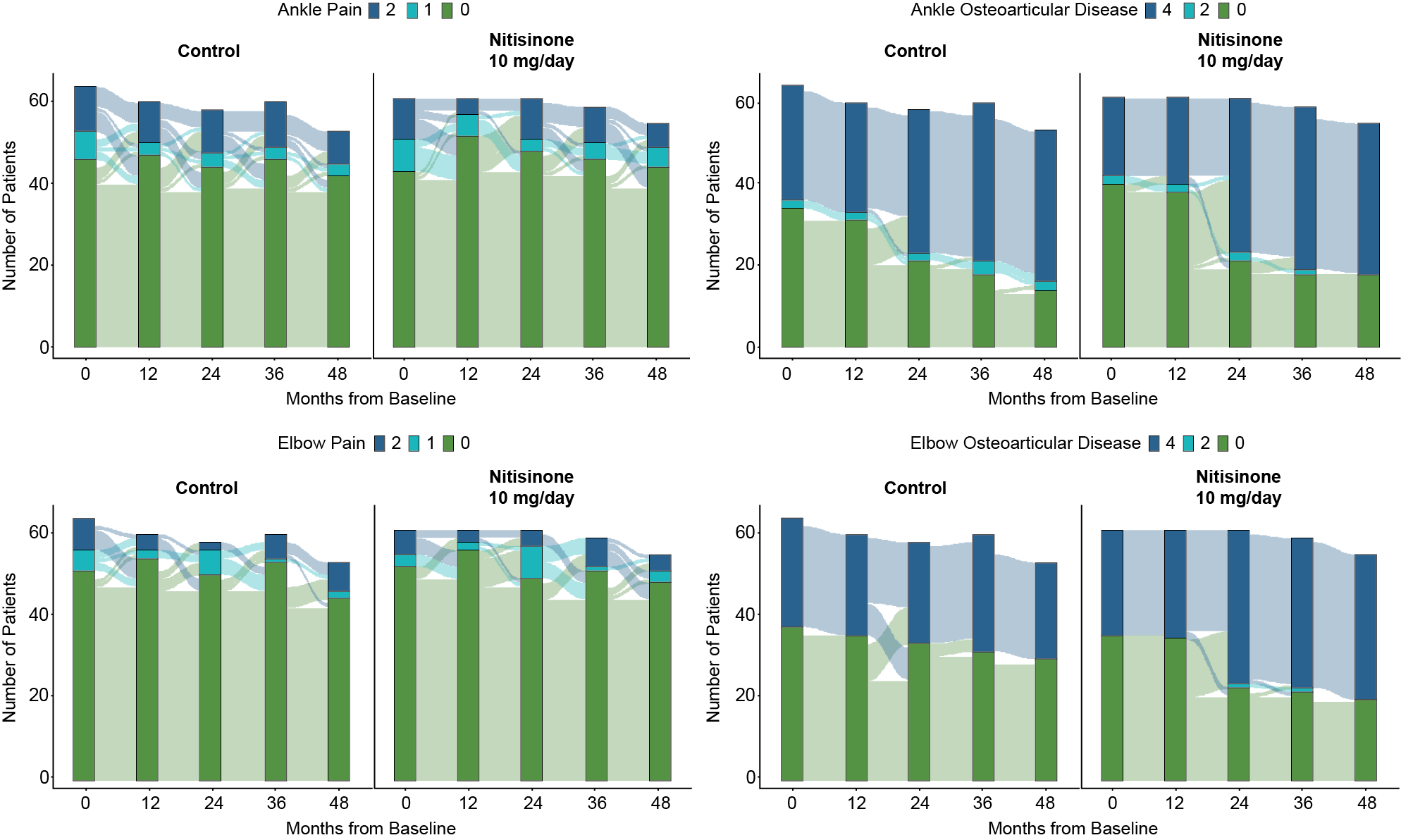


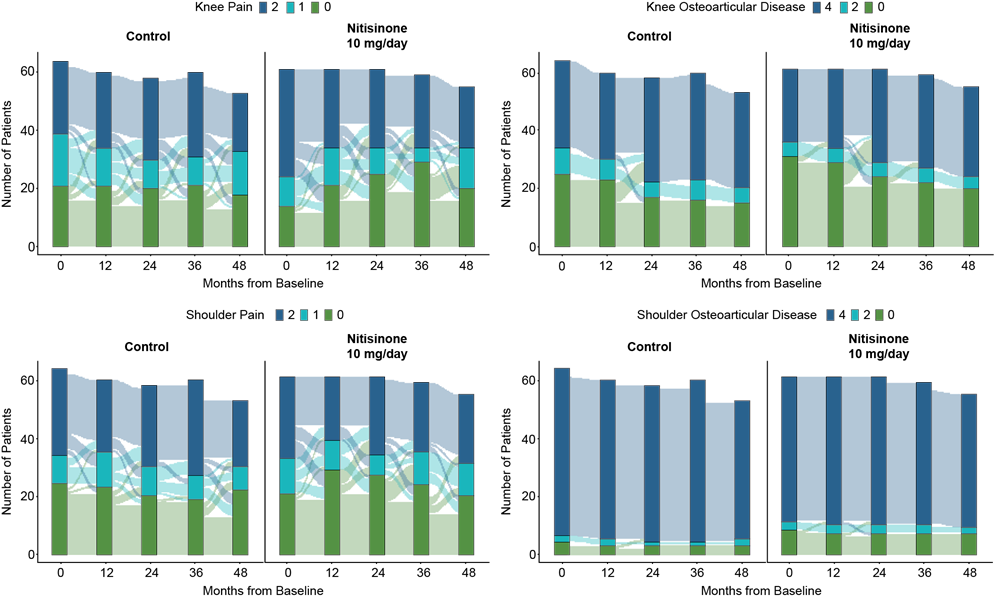


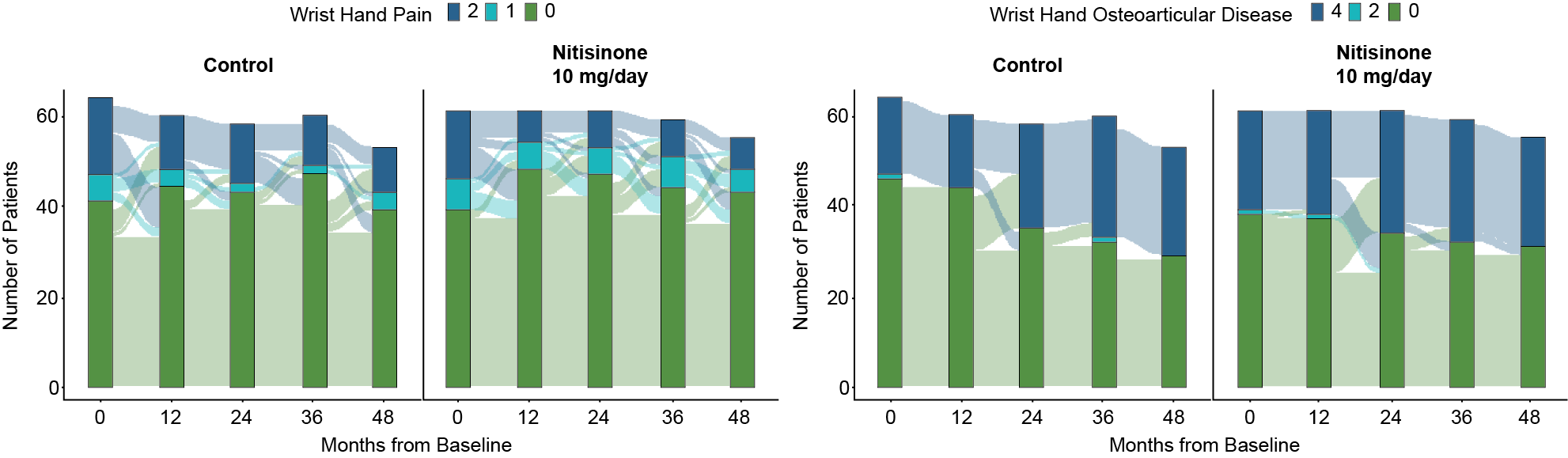


AKUSSI: Alkaptonuria Severity Score Index.

Supplementary Figure 4: Alluvial plots showing changes in spine rheumatology features of the AKUSSI score for components over time, nitisinone vs control


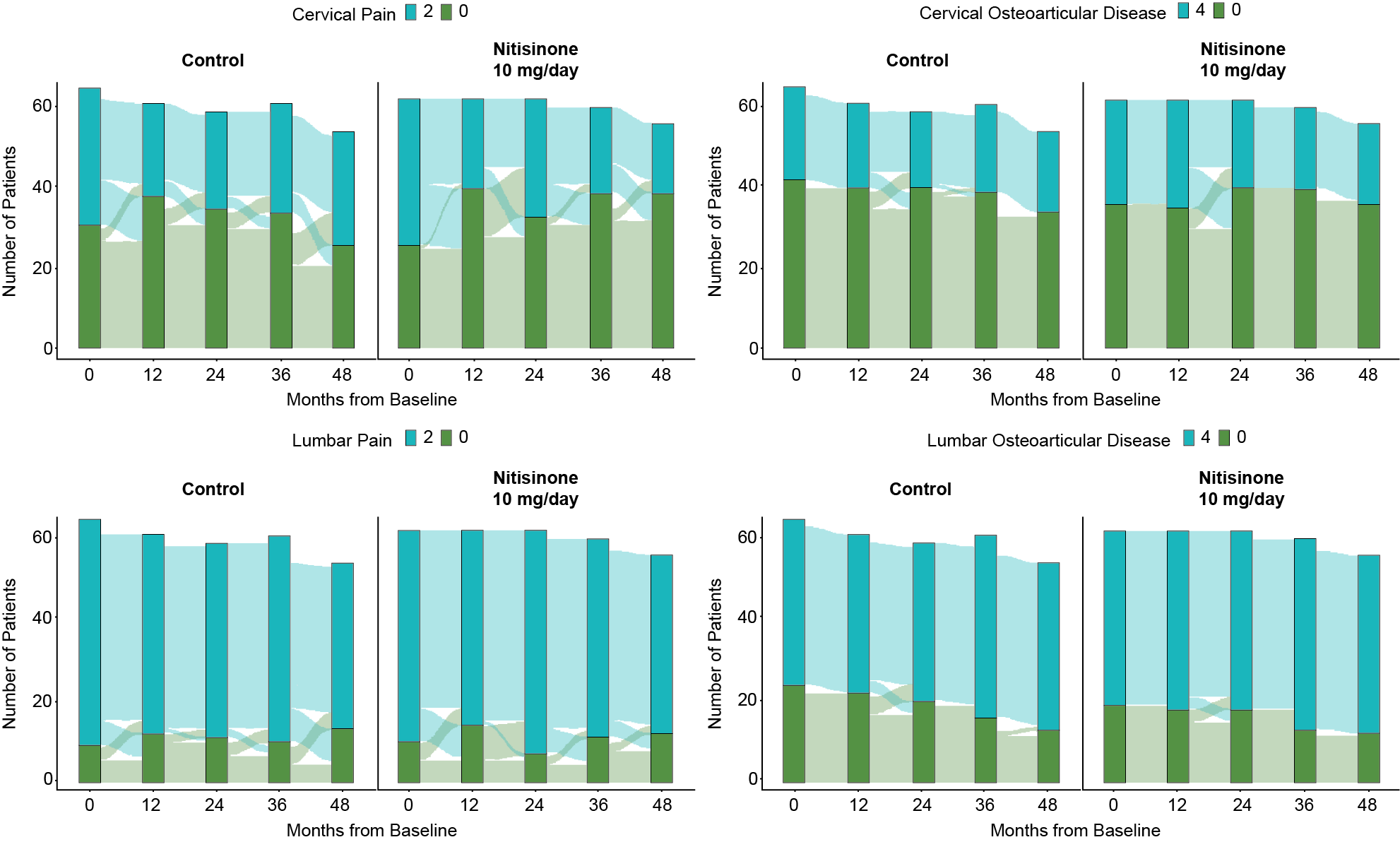


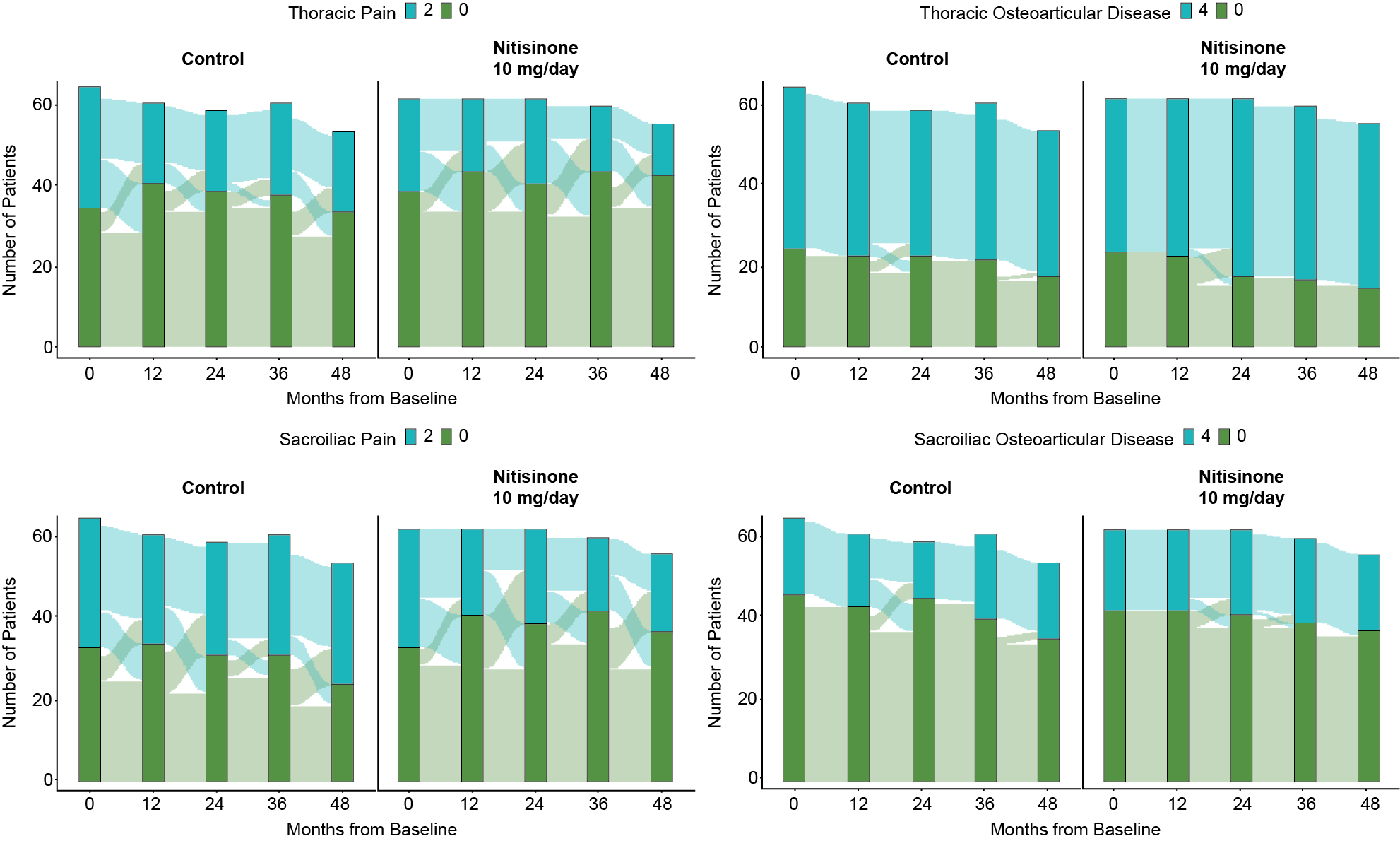


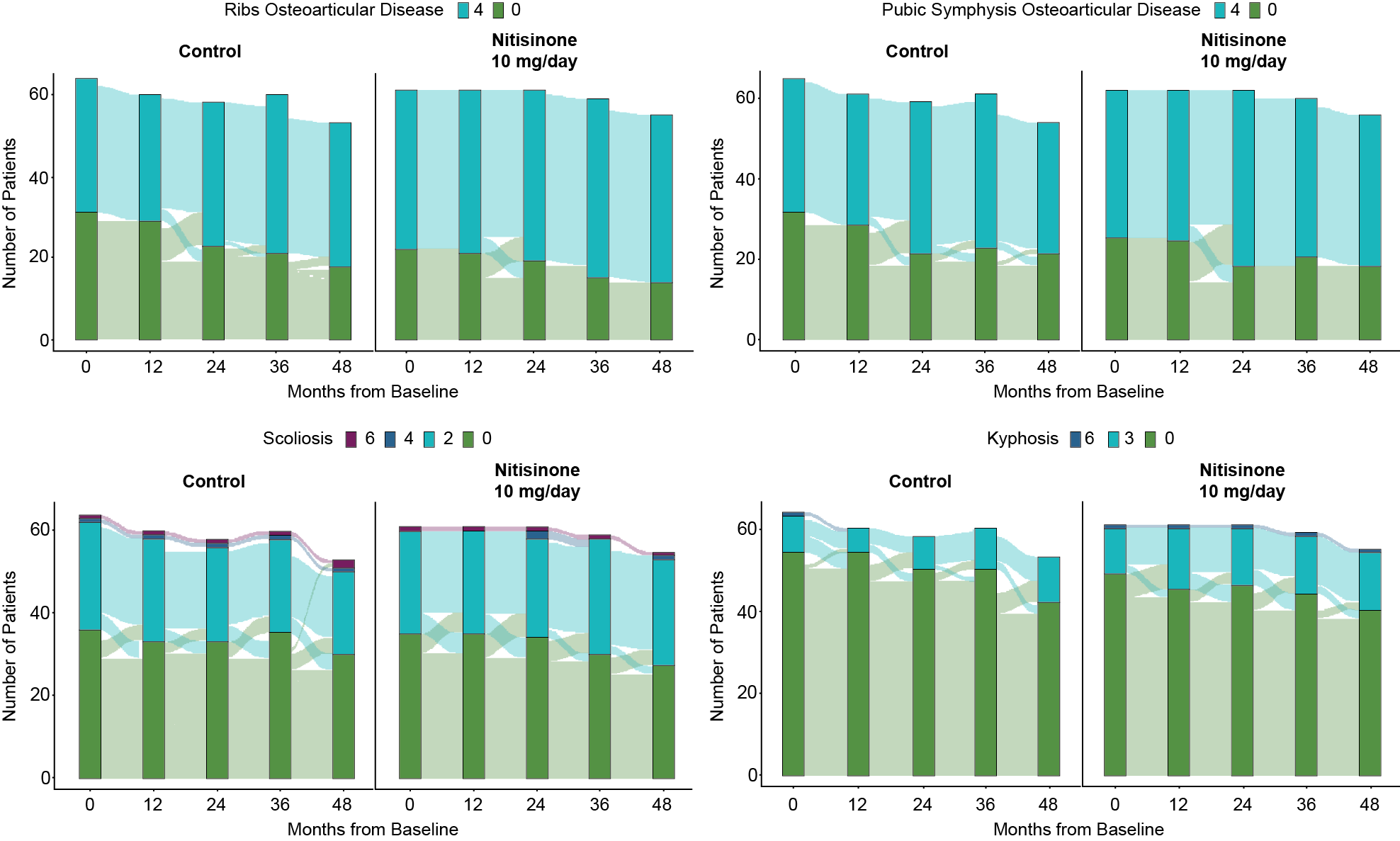
AKUSSI: Alkaptonuria Severity Score Index.

## Flex-AKUSSI

Supplementary Figure 5: Change from baseline over time for the flex-AKUSSI* removing each piece of resource-intensive equipment and the cAKUSSI, nitisinone vs control


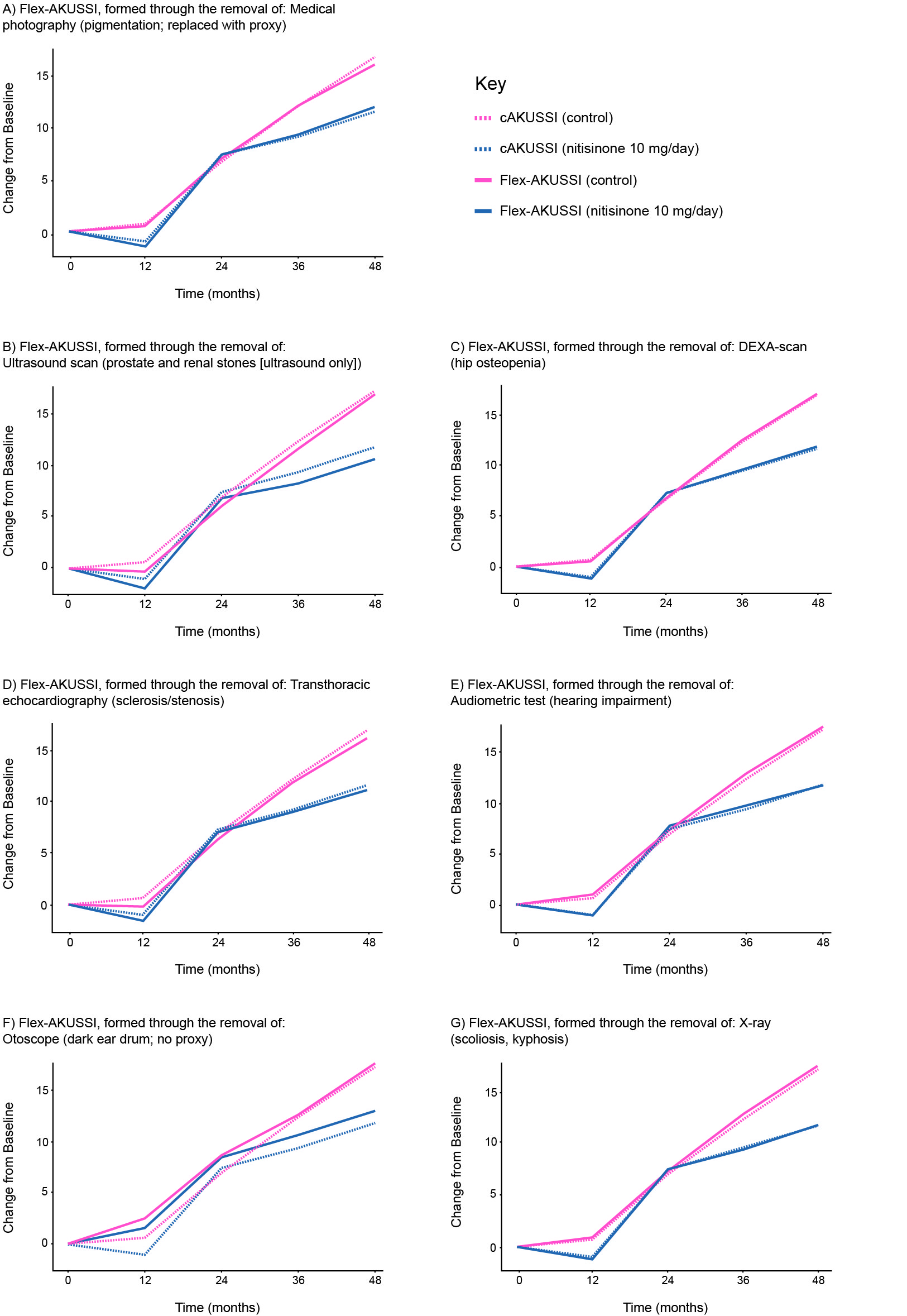


*Flex-AKUSSI scores were adjusted to be on the same scale as the cAKUSSI. cAKUSSI: clinical evaluation Alkaptonuria Severity Score Index; DEXA: dual energy X-ray absorptiometry.

## Contribution of osteoarticular disease

Supplementary Table 6: Osteoarticular disease cAKUSSI contribution measured using PET-CT/Tc99m MDP scans, change from baseline

| **Visit** | **Control,**  **Mean (SD)** | **Nitisinone,**  **Mean (SD)** |
| --- | --- | --- |
| Month 12 | -0.03 (0.26) | 0.89 (6.18) |
| Month 24 | 3.48 (7.72) | 5.25 (10.3) |
| Month 36 | 5.07 (8.69) | 6.68 (11.1) |
| Month 48 | 5.13 (9.09) | 7.16 (11.0) |

cAKUSSI: clinical evaluation Alkaptonuria Severity Score Index; PET-CT: positron emission tomography–computed tomography; SD: standard deviation; Tc99m MDP: technetium-99m methyl diphosphonate.

REFERENCES

1. Cox TF, Ranganath L. A quantitative assessment of alkaptonuria: testing the reliability of two disease severity scoring systems. Journal of Inherited Metabolic Disease: Official Journal of the Society for the Study of Inborn Errors of Metabolism 2011;34:1153-1162. <https://doi.org/10.1007/s10545-011-9367-8>

2. Ranganath LR, Psarelli EE, Arnoux J-B, et al. Efficacy and safety of once-daily nitisinone for patients with alkaptonuria (SONIA 2): an international, multicentre, open-label, randomised controlled trial. The Lancet Diabetes & Endocrinology 2020;8:762-772. <https://doi.org/10.1016/S2213-8587(20)30228-X>

3. R Core Team. R: A language and environment for statistical computing. R Foundation for Statistical Computing, Vienna, Austria, 2020. <https://www.R-project.org/>.

4. Brunson J, Read QD. ggalluvial: Alluvial Plots in 'ggplot2'. R package version 0.12.3., 2020.

5. Wickham H. ggplot2: Elegant Graphics for Data Analysis., 2016.
